# Parametric Design Studies of Mass-Related Global Warming Potential and Construction Costs of FRP-Reinforced Concrete Infrastructure

**DOI:** 10.3390/polym14122383

**Published:** 2022-06-12

**Authors:** Philipp Preinstorfer, Tobias Huber, Sara Reichenbach, Janet M. Lees, Benjamin Kromoser

**Affiliations:** 1 Department of Engineering, University of Cambridge, 7a JJ Thomson Ave, Cambridge CB3 0FA, UK; jml1010@cam.ac.uk; 2 Institute of Structural Engineering, TU Wien, Karlsplatz 13/212-2, 1040 Vienna, Austria; tobi.huber@tuwien.ac.at; 3 Institute of Green Civil Engineering, University of Natural Resources and Life Sciences, Peter-Jordan-Straße 82, 1190 Vienna, Austria; sara.reichenbach@boku.ac.at (S.R.); benjamin.kromoser@boku.ac.at (B.K.)

**Keywords:** FRP reinforcement, FRP-reinforced concrete, global warming potential, application potential, textile reinforcement, parametric design, concrete infrastructure, sustainable infrastructure buildings

## Abstract

Fibre-reinforced polymers (FRPs) are a promising corrosion-resistant alternative to steel reinforcement. FRPs are, however, generally costly and have a high energy demand during production. The question arises whether the high performance of FRPs and possible savings in concrete mass can counterbalance initial costs and environmental impact. In this paper, a parametric design study that considers a broad range of concrete infrastructure, namely a rail platform barrier, a retaining wall and a bridge, is conducted to assess the mass-related global warming potential and material costs. Design equations are parametrised to derive optimum reinforced concrete cross-sectional designs that fulfil the stated requirements for the serviceability limit state and ultimate limit state. Conventional steel reinforcement, glass and carbon FRP reinforcement options are evaluated. It is observed that the cross-sectional design has a significant influence on the environmental impact and cost, with local extrema for both categories determinable when the respective values become a minimum. When comparing the cradle-to-gate impact of the different materials, the fibre-reinforced polymer-reinforced structures are found to provide roughly equivalent or, in some cases, slightly more sustainable solutions than steel-reinforced structures in terms of the global warming potential, but the material costs are higher. In general, the size of the structure determines the cost competitiveness and sustainability of the FRP-reinforced concrete options with the rail platform barrier application showing the greatest potential.

## 1. Introduction

A large part of existing infrastructure is built out of reinforced concrete. To protect the internal steel reinforcement from corrosion, a suitable concrete cover (20–60 mm) is required. A loss of passivation to the steel over time due to progressive carbonation of the concrete or the ingress of chlorides can lead to the corrosion of the reinforcement and the deterioration of the structure [1]. A possible solution is the implementation of fibre-reinforced polymer (FRP) reinforcement. The corrosion resistance of FRPs can not only increase the durability of a structure but also increase resource efficiency due to a reduction in the concrete cover. Additionally, FRP reinforcement is characterised by a low density and a high tensile strength. This can lead to advantages from both an economic [2,3] and environmental point of view [4].

The first investigations regarding the implementation of FRPs in structural concrete took place in the 1960s [5,6,7,8,9]. The research was brought back into the spotlight at the beginning of the twentieth century with the development of textile FRP reinforcement [10,11,12], resulting in pilot projects [13,14,15,16] to demonstrate the technical feasibility and potential. Numerous national guidelines [17] and standards [18] as well as international association recommendations [19] are readily available for the design and construction of FRP-reinforced structures. Higher initial costs [2,20], however, have been articulated as barriers to more widespread application. But on the aspired path to climate neutrality and in view of emerging global shortages of raw materials, potential application areas of FRP reinforcement are likely to emerge. Concrete infrastructure applications hold particular promise due to (i) long service lives, (ii) stringent demands for material-efficient design and (iii) a desirability for minimal in-service maintenance requirements [21].

A longer service life is anticipated due to the corrosion resistance of FRPs, which also lowers future maintenance costs. Corrosion-related damages are the main cost drivers for steel-reinforced concrete infrastructure [22,23,24,25]. Hence, the avoidance of corrosion could justify higher initial costs. However, research on the long-term durability properties of FRP reinforcement is still ongoing, e.g., [26,27,28,29]. Several factors influence the durability of these reinforcements, such as stress, temperature, moisture and alkalinity, with stress having the greatest impact [19]. The dependency on these factors differs for the respective FRP reinforcement product (fibre material, matrix material and product shape). An overview of typical products is given in [30] where carbon fibre-reinforced polymer (CFRP) reinforcement was generally found to exhibit the best durability properties. Nevertheless, an increase in service life can still be expected, and indeed, an early example of an FRP-reinforced concrete bridge was assessed after more than 15 years of service and showed no significant degradation [31]. 

**Figure 1:**
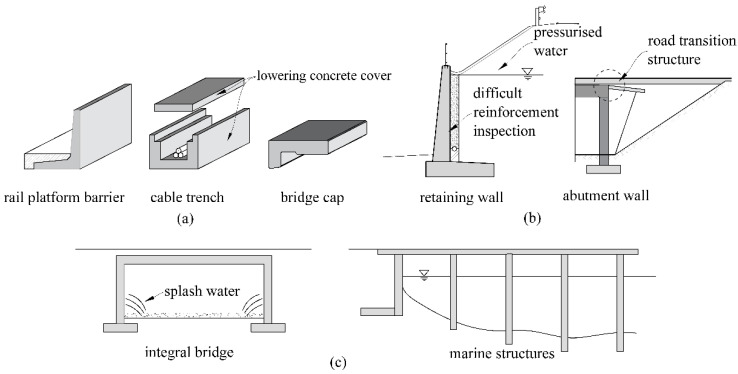
Potential of FRP application for concrete infrastructure: (**a**) small-sized, (**b**) medium-sized and (**c**) large-sized structures.

Structures subjected to harsh environments are therefore likely to benefit most from the application of FRP reinforcement. Such structures include (see Figure 1): (i) structures with de-icing salt exposure (e.g., bridge caps, rail platform barriers; see also Section 3.1), (ii) splash water areas in road infrastructure (e.g., support walls in overpasses; see also Section 3.3, inner shells of tunnels), (iii) areas of pressurised water (e.g., behind abutment walls due to consequential damage from leaky road transition structures, behind retaining walls; see also Section 3.2) or (iv) marine structures [32]. Furthermore, the potential enhancement of material efficiency due to the inclusion of FRP textile reinforcement could be exploited (i) in mass-produced thin elements due to concrete cover reduction (e.g., cable trenches, cable trench cover plates, rail platform barriers, Figure 1c) or (ii) by utilising the high tensile capacity of FRPs for a slim design of highly reinforced, critical components (e.g., bridge supports or frame corners at integral bridges; see also Section 3.3). The use of FRP reinforcement could also enhance the maintenance process by using the conduction of CFRPs to provide full-surface-humidity monitoring, with the option of preventive cathodic corrosion protection [33]. This novel approach could be highly relevant for critical steel reinforcement layers that cannot be inspected and contribute to the load-bearing capacity (e.g., behind retaining walls, in bridge decks). Fire safety is paramount and the fire resistance of FRPs is an active area of research, e.g., [34,35]. However, for the concrete infrastructure applications summarised here, the fire requirements are typically less stringent than would be required in buildings, thereby removing a major barrier for the application of FRP reinforcement. 

As stated before, FRPs are usually quoted as being expensive based solely on the initial cost of the product. More holistic investigations that consider potential material savings and the overall environmental footprint should take into account various parameters such as the structure size. However, to date, such investigations are rarely reported in the literature. This paper seeks to identify application areas with the greatest potential for realising the benefits of FRP reinforcement. A parametric design study is undertaken where the concrete mass and weight of the main longitudinal reinforcement are linked to characteristic values of global warming potential (GWP) and costs. The investigation covers a broad range of structures that are representative of small (rail platform barrier) to medium (retaining wall) and large (bridge) structures exposed to harsh environments. The costs and GWP of the concrete and steel or FRP reinforcement (focusing on the main longitudinal reinforcement) over stages A1–A3 (cradle to gate; A1: Raw Material Supply, A2: Transport and A3: Manufacturing) of an LCA analysis are compared. The results serve as the foundation for an evaluation of not only the types of structures that could benefit most from FRP reinforcement alternatives but also structures whereby FRPs may not be currently competitive on the basis of the A1–A3 cost or environmental metrics. 

## 2. Materials and Methods

### 2.1. Materials

In addition to ordinary steel reinforcement, this study investigates FRP reinforcement with different fibre materials, namely carbon (CFRP) and glass (GFRP) in the form of bars (GFRP and CFRP) or textiles (only CFRP). Other materials such as basalt (BFRP) are also suitable, but concerns about the durability of basalt in the alkalinity of the concrete remain [36,37]. Although, however, BFRP is not investigated in this study, its properties are similar to GFRP reinforcement, thus the general conclusions are likely to be analogous. The long-term tensile strength *f*_tk,100_ is calculated according to ACI 440.1R-15 [38] considering environmental (factor *C*_E_) and temperature aspects (factor *C*_T_), see Equation (1). In the case of GFRP, which is not as resistant as carbon, more onerous reduction factors would apply.

(1) $$f_{\mathrm{tk},100}=C_{T}\cdot C_{E}\cdot f_{\mathrm{tk}}$$

The design strength *f*_td_ is calculated as the long-term strength *f*_tk,100_ divided by the material safety coefficient *γ*_m_. For FRP rod reinforcement, the safety coefficient was taken as 1.5, whereas for textile reinforcement, a smaller value of 1.3 was used in accordance with recent investigations [39]. The bond strength, which is important for the crack-width calculation, is taken to be of same magnitude as the steel reinforcement [40,41]. The input values of the FRP reinforcement are summarised in Table 1.

The selected B550B steel reinforcement is commonly used in Austria with a characteristic yield strength *f*_yk_ of 550 MPa and Young’s modulus of *E*_s_ = 200 GPa. In terms of the concrete, different strength classes ranging from C20/25 to C50/60 are considered in this study. All the necessary input material properties for the steel and concrete are taken from EN 1992-1-1 [45] and are shown in Table 2. Creep effects are considered using a creep value *φ*_(t0,t∞)_ = 2.0. To reflect a harsh environment, the exposure class is taken to be higher than XC2, meaning that the allowable crack width for steel reinforcement is limited to 0.3 mm under quasi-permanent loads, while it is 0.5 mm for the FRP-reinforced variants in accordance with ACI 440.1R-15 [38]. Differences also arise in the determination of the concrete cover thickness, where for the FRP reinforcement solely the minimum cover *c_b_* for bond in dependency of the reinforcement diameter *c*_b_ = 2 · *Ø*_r_ was taken. For the steel reinforcement, additional durability criteria according to EN 1992-1-1 [45] has to be fulfilled. Finally, the concrete density *ρ* is consistently taken as 2.4 t/m³, while the concrete material safety coefficient is *γ*_m_ = 1.5.

### 2.2. Methods

#### 2.2.1. Design Provisions

The design of structures is governed by various requirements in the serviceability limit state (SLS) and ultimate limit state (ULS). The following requirements are considered in the current work: (a) flexural capacity, (b) deflection control, (c) crack-width control and (d) stress limitation. In the parametric comparisons, the main longitudinal reinforcement requirements are calculated but any shear reinforcement is not taken into account. The structures examined, however, are not shear sensitive. 

In recent decades, various guidelines and frameworks have been developed for the design, dimensioning and testing of FRP structures (e.g., [18,19,38,46,47,48,49,50]) with a comprehensive overview given by Emparanza et al. [51]. The North American guidelines are regularly updated and a new annex dealing with the design of concrete structures reinforced with FRP is planned for European regulations (EC2) [52]. The guidelines, however, mainly focus on bar reinforcement and do not reflect the current developments in the field of textile reinforcement [30]. The first European guideline that includes both FRP bar reinforcement and textile reinforcement is therefore currently being developed [11]. For current applications of FRP in reinforced concrete structures, however, a building product approval or approval in individual cases is in many cases still needed [53]. In the work presented here, the structures were designed according to the EN 1992-1-1 [45] regulations for reinforced concrete with due consideration of the regulations in ACI 440 [38] in the case of FRP-specific requirements. As FRP reinforcement typically has a higher tensile strength but a lower stiffness, the influence of these differences on the design is additionally studied in Appendix A.

**Flexural Capacity:** As FRP reinforcement does not yield, the flexural failure of an FRP-reinforced structure can either be governed by the rupture of the reinforcement or a concrete compression failure. The flexural capacity can be determined by varying the longitudinal strain distribution through the depth of the section until an equilibrium of horizontal forces is reached. If, however, some assumptions are made, a closed-form solution can be derived, which simplifies the design procedure. In Rempel et al. [54], for example, such a closed-form solution based on a stress block in the concrete compression zone was derived. The equations which are also used for the parametric study are given in Annex A.1, together with an evaluation of the influence of the modulus ratio *α*_r_ = *E*_r_/*E*_c_ and the strength ratio *μ = f*_td_*/f*_cd_ of the FRP reinforcement and concrete, which differs for different FRP reinforcement products, on the flexural capacity.

**Deflection Control:** The calculation of the deflections by numerical integration of the curvature *κ* along the beam has been found to be in good agreement with the actual deflections, also for FRP-reinforced structures [55]. The partly cracked stage is considered according to EN 1992-1-1 [45] with a distribution coefficient *ζ* between uncracked *κ*_I_ and fully cracked *κ*_II_ stage. The deflection at specific points can be calculated using the principle of virtual deflection with section-wise-determined curvatures *κ*_m_. Effects of creep and shrinkage can be included in the calculation of the curvature. The corresponding equations are given in Appendix A.2, together with an evaluation of the influence of the modulus ratio *α*_r_ = *E*_r_/*E*_c_ and strength ratio *μ = f*_fd_*/f*_cd_ of the FRP reinforcement and concrete. For smaller reinforcement ratios *ρ* and smaller strength ratios *μ*, the feasible *l/d* ratio potentially gets larger as the loading derived from the ultimate flexural capacity decreases. The modulus ratio *α*_r_, however, only plays a role for higher values of *ρ*_l_, where a bigger section of the beam is in a cracked state.

**Crack-width control:** The crack width in reinforced structures corresponds to the integrated strain differences between reinforcement *ε*_r_ and concrete *ε*_c_ along the crack distance *s.* The maximum crack distance corresponds to two times the transfer length. A constant bond stress *τ*_m_ along the transfer length is assumed in the following. In the case of the stabilised crack pattern, the crack width can eventually be calculated using the tension beam analogy [56]. The corresponding equations are given in Appendix A.3, together with an evaluation of the influence of the modulus ratio *α*_r_ = *E*_r_/*E*_c_ and different stress levels in the reinforcement. The Young’s modulus of elasticity and the stress in the reinforcement directly influence the crack width. The elongation stiffness of FRP reinforcement is typically less than that of steel and this leads to larger crack widths. Because FRP reinforcement, however, is not prone to corrosion, the allowable crack width in such structures can be generally higher compared to steel-reinforced structures. This is, for example, recognised by ACI 440.1R-15 [38], which allows crack widths between 0.4–0.7 mm (see also Section 2.1). 

**Stress limitation:** Considering a triangular stress distribution in the concrete compression zone in the SLS, the compression zone height and the maximum stresses in the concrete σ_c_ and the reinforcement σ_r_ can be calculated. The corresponding equations are given in Appendix A.4, together with an evaluation of the influence of the modulus ratio *α*_r_ = *E*_r_/*E*_c_. A strong dependency of the reinforcement ratio on the stresses can be observed for both the concrete and the reinforcement, whereas the modulus ratio primarily influences the concrete stresses. Under critical serviceability load conditions, the stresses usually have to be limited in (a) the concrete to avoid longitudinal cracking of the concrete (factor *k*_1_ in EN 1992-1-1) and nonlinear creep effects (factor *k*_2_ in EN 1992-1-1) and (b) the reinforcement, where the steel reinforcement stress is limited to avoid yielding and excessive crack opening (factor *k*_3_ in EN 1992-1-1), while in FRP reinforcement, the reinforcement stresses are limited to avoid creep rupture (factor *C*_C_, see Section 2.1). In the case of CFRP, higher stress limits are allowed due to the better material properties.

#### 2.2.2. Parametrisation of Design

**Figure 2:**
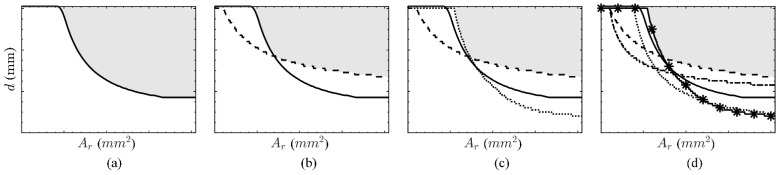
Optimum design to meet the requirements for the (**a**) ULS flexural capacity (continuous line), (**b**) ULS + SLS deflection control (dashed line), (**c**) ULS + SLS deflection control + SLS crack-width limitation (dotted line), (**d**) ULS + SLS deflection control + SLS crack-width limitation + SLS stress FRP and concrete stress limitation (dash-dotted and asterisk marked line), where the feasible combinations of cross sections and reinforcement areas are indicated as a grey area [57].

The provisions in Appendix A show that the design of reinforced concrete structures depends on various parameters, such as the modulus and strength ratio of reinforcement and concrete, and that these differ between steel and FRP reinforcement. Hence, a direct comparison of individual designs with fixed cross-sectional dimensions or reinforcement ratios is not suitable, but rather, an optimum design for each type of reinforcement can be more revealing. The design provisions described previously are therefore parametrised by the effective depth *d* and the reinforcement cross-sectional area *A*_r_ to determine optimum design curves [3] using a ‘brute force method’, where: (a) the reinforcement ratio is increased in stepwise increments, (b) for each increment the effective depth is varied within a specified range and the limit state of interest is calculated for each step (e.g., the flexural capacity) and (c) the minimum effective depth to fulfil an individual requirement (e.g., acting design bending moment < flexural capacity) is assigned to the given reinforcement ratio. This creates a design curve, where values above this curve describe the feasible zone of *A*_r_/*d* combinations; see, for example, Figure 2a, where the *A*_r_/*d* values that fulfil the flexural capacity of a representative concrete cross section are given by the grey shaded area above the design curve. The optimum design curve is eventually described by the *A*_r_/*d* ratio at which all investigated requirements in SLS (deflection control, stress limitation, crack-width limitation) and ULS (flexural capacity) are fulfilled [57]. The feasible zone may decrease with each SLS or ULS requirement, see Figure 2b,c. Additionally to the crack-width limitation, a minimum reinforcement area is necessary that can take the tensile stresses at cracking of the concrete. This leads to an effective depth limit for every specific reinforcement area and therefore cuts off the feasible area from the top. This requirement is checked in the following parametric study but, for the sake of clarity, not displayed in the diagrams. 

#### 2.2.3. Economics and Environmental Considerations

It is possible to obtain a direct link between the structure’s environmental impact in terms of the global warming potential (GWP) and the presented optimum design functions by multiplying the reinforcement and concrete masses of each *A*_f_ and *d* combination along the optimum curve by the environmental impact per m³ or kg, respectively. The values per mass based on a cradle-to-gate life cycle assessment were extracted from the literature [4] and are presented in Table 1 and Table 2. The presented data include the environmental impact of the production phase (A1–A3 according to [58]) with a declared unit of 1 kg of the reinforcement product and one cubic meter of concrete. The GWP of the steel reinforcement is based on basic oxygen steelmaking (BOS). Other methods such as electric arc furnace (EAF) have a lower environmental impact, especially if green energy is used. Because BOS, however, accounts for about 2/3 of the steelmaking in Europe [4], GWP of steel that is related to this production method was chosen for this study. Different environmental impact values for the materials under consideration would lead to different conclusions, but nevertheless, the framework for relative evaluation would remain applicable. 

Furthermore, the optimised design functions can also be linked to economic data. The costs per m³ or per kg, respectively, are presented in Table 1 and Table 2. It should be noted that a broad range of products are available within Europe and several factors influence the price of FRPs, such as the quantity, transportation costs and the desired product [30]. For this study, data taken from the German-speaking market (FRP: [43,44]) are chosen, as they already have or at least are close to having technical approval in Germany. Moreover, the prices considered in this study do not reflect world crises since 2019. The steel price, in particular, has increased significantly recently. The volatile market situation and the influence on the results will be discussed in more detail in Section 4.1.2.

The mass-related connections to economic or environmental aspects enable a comparison of the efficiency of structures reinforced with different types of reinforcement. This is performed in the following for both the economic as well as the environmental impact in terms of the GWP. An overall efficiency that reflects both cost and environmental aspects can be assessed, but weighting factors must be defined for each impact category. These factors can differ for various projects. It is also expected that environmental impact categories will become more important in the future, whereas in current practice, the cost is often the dominant decision criterion. Hence, for the sake of clarity, both impact categories are studied individually in this paper. The assessment at this stage of the investigations is based solely on the material masses. Other impacts that arise, for example, from the formwork, are considered to be the same for all reinforcement products and are therefore omitted in the results in Section 3. Even though this is a simplification, it is justifiable in the context of this study, which generally seeks to identify types of structures that could benefit most from FRP reinforcement alternatives. With the same reasoning, no structural reinforcement except for the main longitudinal reinforcement is included in the comparison of the individual variants. Concrete infrastructure of different sizes, ranging from very small- (rail platform barriers) to medium- (retaining walls) and large-size structures (integral bridges), are investigated. A further point of note is that the focus is on A1–A3 stages, and an LCA assessment of A4–A6, B1–B5, C1–C4 and D life cycle stages will be addressed in further studies, particularly because no comprehensive data on the entire life cycle of FRP reinforcement is currently available. Indeed, such an evaluation would probe additional factors such as the possible extension of service life due to the FRP reinforcement’s high corrosion resistance [59,60].

## 3. Results

### 3.1. Small-Size Structures

Small-size structures in concrete infrastructure are common and varied. These include components without major requirements in the SLS and ULS, such as cable trenches. In this study, L-shaped rail platform barriers are investigated. The walls of such structures typically have a length of 0.95 m in Austria, according to general design provisions of the Austrian Railway operators (ÖBB). Usually, a concrete class C25/30 is used. The barrier is loaded by earth pressure due to the backfilling, and additional loadings arise from crowds on the platform and a service car for maintaining the platform. The exact loading conditions are provided in Appendix B.1. For the parametric study, the vertical wall of the barrier is modelled as a cantilever, with the thickness of the wall being assumed to be constant over the length and of the same magnitude as the slab thickness. In the medium- and large-size structures described in Section 3.2 and Section 3.3, FRP bars are assumed. However, the smaller size of the rail platform barriers allows for the usage of textile CFRP reinforcement with its advantageous mechanical performance and lower costs compared to FRP bars. Therefore, in this particular example, the concrete cover thickness is set to 10 mm in the case of the CFRP textile, while for the GFRP bar- and steel-reinforced structure, the concrete cover is set to 20 and 35 mm, respectively.

**Figure 3:**
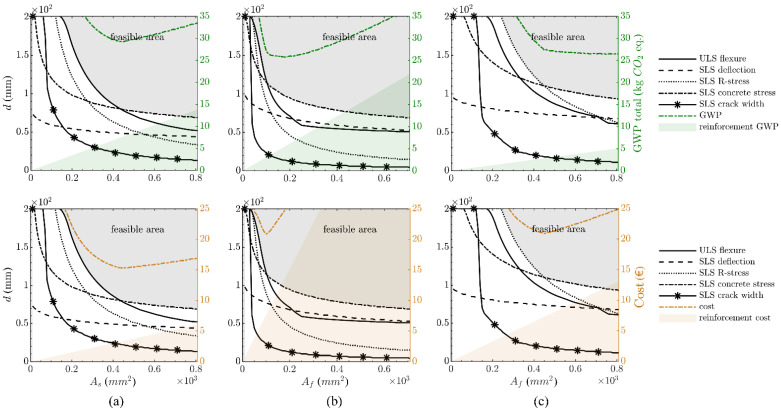
Optimum design of rail platform barrier (per one-metre length): steel-reinforced structure (**a**), CFRP textile-reinforced structure (**b**) and GFRP bar-reinforced structure (**c**), with consideration of GWP (top) and costs (bottom).

The results of the parametric study are depicted in Figure 3. It is observed that for the steel-reinforced structure, the ULS flexural capacity typically governs the structural dimensions except for very high reinforcement areas, while for the FRP-reinforced cases, SLS stress limitations control. Crack-width control does not play a significant role in this example due to the low quasi-permanent loads. In the case of the CFRP, the design is generally governed by the concrete stresses, while for the GFRP, the reinforcement stresses determine the structural thickness for small reinforcement areas, whereas the concrete stresses control in the case of a larger reinforcement area.

When the material mass of the solutions that bound the optimum design curves is linked to the GWP and the costs (green and orange dotted line in Figure 3), significant differences become evident: in the case of steel- and CFRP-reinforced structures, a local minimum both for the cost and the GWP is ascertainable, being, however, more pronounced in the case of CFRP-reinforced structures. This means that an optimum design is essential to achieve a cost-efficient and more sustainable solution. Note that the costs are ‘cut off’ for higher reinforcement ratios in Figure 3b as they are significantly higher compared to the other variants. For the sake of comparability, however, the same scale was used for the y-axis in all cases. For the GFRP-reinforced structure, the local minimum is also ascertainable in the costs, but the GWP progressively decreases with an increasing reinforcement area. This means that the rising GWP due to the larger reinforcement area is offset by the GWP decrease through a reduction in concrete mass, leading to progressively more sustainable solutions.

Based on the mass-related GWP calculations, it is observed that when compared to steel reinforcement (29.2 kg CO_2_ eq/m), both the CFRP (25.7 kg CO_2_ eq/m; −11.9%) and GFRP (26.4 kg CO_2_ eq/m; −9.7%) options provide somewhat more sustainable solutions. However, the opposite trend is apparent for the costs, with the RC structure (15.3 EUR/m) exhibiting the lowest costs when compared to the CFRP- (20.9 EUR/m; +36.7%) and GFRP-reinforced (20.9 EUR/m; +36.7%) structures.

### 3.2. Medium-Size Structures

An L-shaped structure is also taken to be representative of medium-sized structures in concrete infrastructure, but in this instance, a retaining wall is considered as an example. L-shaped retaining walls are commonly used to secure height differences in terrain. Hence, they tend to have a greater wall height than railway barriers and a higher loading that predominantly arises from the earth pressure behind the wall. The loading conditions used in the parametric study are given in Appendix B.2. Following the procedure detailed in the previous section, only the design space for the vertical wall element is calculated. However, the relative comparison of the wall solutions also enables a qualitative comparison of the whole structure. The wall length is taken as 5 m, which is a representative length for a medium-sized retaining wall. Typically, a concrete class C25/30 is considered. Due to the expected larger dimensions of the structure, the CFRP bars with larger reinforcement areas instead of textiles are used. The reinforcement in all variants is placed in a single layer. Reinforcement bars with a diameter of 10 (FRP) and 16 mm (steel) are used, leading to a concrete cover thickness of 20 and 40 mm in the case of the FRP- and steel-reinforced structure, respectively.

**Figure 4:**
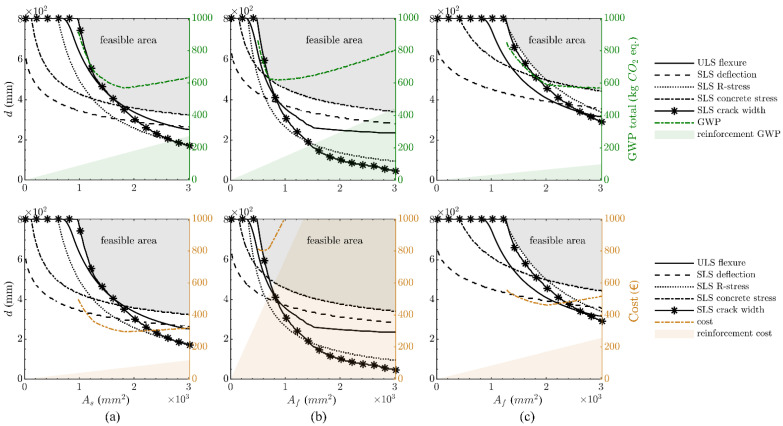
Optimum design of retaining wall (per one-metre length): steel-reinforced structure (**a**), CFRP-reinforced structure (**b**) and GFRP-reinforced structure (**c**), with consideration of GWP (top) and costs (bottom).

The design results are shown in Figure 4 for the reinforcement materials under consideration. The optimum design curve in the case of the FRP combinations is again driven by the SLS stress limitations, whereas for the steel-reinforced structure, it is governed either by the crack-width limitation or flexural capacity. Only in the case of large reinforcement areas is the design governed by the concrete stress limitation (in the absence of compressive reinforcement).

In terms of the GWP, the relative advantage of the FRP reinforcement observed in the small size example is no longer apparent, with the steel- (569.0 kg CO_2_ eq/m) and GFRP-reinforced (567.2 kg CO_2_ eq/m; −0.3%) structures exhibiting almost the same GWP, while the CFRP-reinforced structure (617.3 kg CO_2_ eq/m; +8.5%) has a slightly higher GWP. In terms of the economics, the higher costs that were observed for the FRP-reinforced rail platform barrier are even more apparent for the retaining wall, with the steel-reinforced structure showing a mass-related cost of 295.2 EUR/m, which is significantly lower than the GFRP- (461.4 EUR/m; +56.3%) and the CFRP-reinforced (801.3 EUR/m; +171.4.0%) comparators.

### 3.3. Large-Size Structures

A slab bridge is investigated as being representative of a large-span structure. The bridge span has an effective length of 15 m and is fixed to the bridge abutment (as in a frame bridge), causing a hogging moment that is almost equal to the sagging moment in the midspan of the bridge. This allows an estimate of the overall longitudinal reinforcement required, even though only the cross section at midspan is investigated in this study. The loading consists of the dead load, earth pressure behind the abutment and live loads due to traffic. Other loadings such as temperature or wind are neglected for this example. The exact dimensions and loading conditions can be found in Appendix B.3. Due to the higher loadings, CFRP or GFRP bars with their larger reinforcement area are used in the bridge design. As a simplification, the reinforcement in all variants is considered to be placed in a single layer. The reinforcement bars have a diameter of 16 (FRP) and 20 mm (steel), leading to a concrete cover thickness of 32 and 40 mm in the case of the FRP and steel-reinforced structure, respectively.

**Figure 5:**
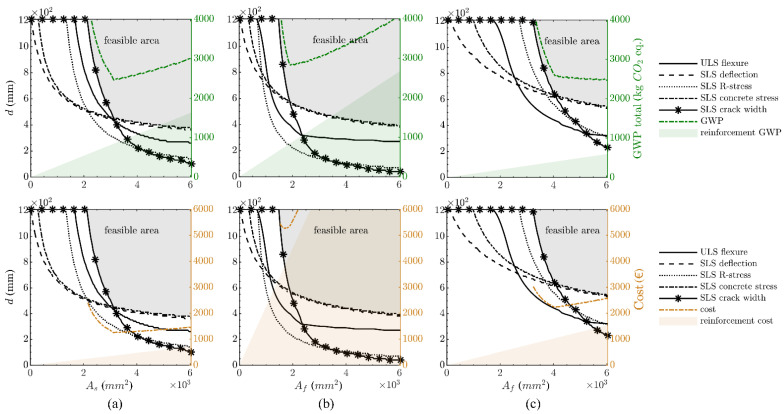
Optimum design of slab bridge (per one-metre length): steel-reinforced structure (**a**), CFRP-reinforced structure (**b**) and GFRP-reinforced structure (**c**), with consideration of GWP (top) and costs (bottom).

The results of the parametric design are depicted in Figure 5. The dimensions of the structures with a low reinforcement area are in all cases governed by the SLS crack-width limitations. For increasing reinforcement areas, the SLS deflection limit becomes critical, although this can in practice be mitigated if the structure is cambered. However, then the concrete stress limitations would start to control. 

When comparing the mass-related GWP for the reinforcement options, similar trends to those in the medium-size structure are evident, with the steel-reinforced structure (2459.0 kg CO_2_ eq/m) showing almost the same mass-related GWP as the GFRP-reinforced structure (2470.0 kg CO_2_ eq/m; +0.4%) and the CFRP structure being slightly higher (2825.3 kg CO_2_ eq/m; +14.9%). In terms of the mass-related costs, the steel-reinforced structure’s costs (1251.6 EUR/m) are significantly lower than the GFRP (2232.6 EUR/m; +78.4%) and CFRP (5275.8 EUR/m; +321.5%) ones. It is noticeable in Figure 5 that, with the exception of the GFRP GWP, the curves for the mass-related GWP and costs exhibit distinct kinks, which describe the local minima. Therefore, it becomes apparent that an optimum design plays a more vital role in larger structures than smaller-sized structures.

## 4. Discussion

### 4.1. Decisive Influencing Parameters

The parametric studies suggest that the environmental performance quantified by calculating the GWP of an FRP-reinforced concrete infrastructure is generally comparable to steel-reinforced structures across the A1–A3 construction stages. In contrast, it was observed that the current construction costs of FRP-reinforced structures are higher. In the following, the most influential parameters underpinning the optimised design and the corresponding mass that drives the GWP and costs will be discussed.

#### 4.1.1. Size of the Structure

In Table 3, the optimum GWP and costs of construction for the structures under investigation are summarised. It is noticeable that the spread in the GWP and costs between steel- and FRP-reinforced structures increases as the size of the structure gets larger. This increase is particularly noticeable in the costs of the CFRP-reinforced structures, which show in terms of large structures such as bridges more than four times (+321.5%) higher mass-related costs than an analogous, optimised steel-reinforced structure. This can mainly be traced back to the significantly higher costs of the CFRP reinforcement needed (+952.1%). A simple replacement of conventional steel reinforcement, therefore, does not seem to be a practical solution, but rather, a different design concept that makes better use of the high-performance but costly and energy-demanding carbon fibres is required. This reaffirms that slender, FRP prestressed structures are a more efficient form of construction [61,62,63,64,65]. For smaller structures, however, the required area of the CFRP reinforcement is relatively small, while a reduction in the concrete cover has a more significant impact on the overall concrete volume. This reduction in material mass leads to more competitive solutions in comparison with steel-reinforced structures.

For GFRP-reinforced structures, the GWP is comparable to that of steel-reinforced structures, with minor savings for small structures, while the costs are moderately higher. Future investigations will probe whether a longer service life justifies higher mass-related costs for the investigated structures.

#### 4.1.2. Reinforcement Type

Not only the type of reinforcement itself (steel or FRP reinforcement) but also the fibre material (carbon or glass) plays a significant role when determining the GWP and costs of a reinforced structure. Table 4 lists the relative contributions of the reinforcement and the concrete on the overall GWP and costs of the individual structures. Because there is a unique optimum for both the GWP and the costs, the share of the concrete and FRP on the overall impact is different for the two categories. It is noticeable that for the FRP variants, the share of the GWP of the reinforcement is lower than for the steel variants, which is particularly the case for the GFRP-reinforced structures. This means that reducing the GWP of concrete would have a greater impact on the overall GWP (see also Section 4.2.1). The opposite picture is seen for the costs, where for the FRP variants, the share of the costs of the reinforcement is much larger than for the steel variants. In the case of the bridge structure, for example, the GWP share of the reinforcement ranges from 34.4 (steel) to 30.1 (CFRP) to 24.1% (GFRP), while the cost share is 29.4 (steel) to 73.4 (CFRP) to 46.7% (GFRP).

The cost of the reinforcement, however, is subject to current and future developments. Considering the recent volatility in the market (2022) due to global crises, the price of steel has increased up to threefold. This sharp increase has not yet been observed for FRP reinforcement where, according to suppliers, the price increase has been about 10–12%. Considering this aspect and the results in Table 4, FRP reinforcement would then be more cost-competitive than it has been in the past. An in-depth study of the influence of market volatility is not conducted here as prices are fluctuating on a daily basis, which hinders definitive conclusions. 

#### 4.1.3. Concrete Compressive Strength

**Figure 6:**
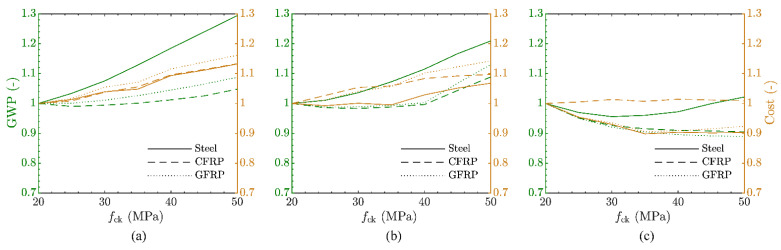
Influence of concrete compressive strength on mass-related GWP and costs of construction (normalised with respect to *f*_ck_ = 20 MPa) of: rail platform barrier (**a**), retaining wall (**b**) and integral bridge (**c**).

It was noticeable, especially in the case of the FRP-reinforced structures, that the material masses were driven by the concrete compressive strength, either by the ULS flexural capacity or by the limitation on the SLS concrete stresses. In this section, the GWP and costs are derived for all three structures using a compressive strength that varied between *f*_ck_ = 20 and 50 MPa, to investigate whether a different concrete strength would lead to a better material utilisation and hence lower costs and environmental impact. The results are shown in Figure 6, where the compressive strength is displayed on the x-axis, while the corresponding normalised GWP and costs are given on the y-axis as green and orange lines, respectively (normalised with respect to the values corresponding to *f*_ck_ = 20 MPa for each variant).

In all cases, a decreasing material mass of the concrete could be observed, usually accompanied by a partial increase in reinforcement mass. A general conclusion, however, on the mass-related GWP and costs cannot be made, but rather, a clear trend for the individual structures and the material mass could be observed. While for smaller-sized structures, an increase in concrete compressive strength leads to a rise in mass-related GWP and costs; this trend progressively shifts for medium and large structures. For the smaller-sized structures, the material savings in concrete mass due to the higher concrete strength therefore do not compensate for the additional reinforcement needed. When comparing the steel- and FRP-reinforced structures, the decrease in mass is more distinct for the FRP-reinforced design than for the steel-reinforced one, leading to a bigger spread between the mass-related GWPs. The higher costs of the FRP reinforcement, however, often counterbalance the decrease in material mass, leading only in the case of larger-size structures to a less expensive structure with increasing compressive strength.

### 4.2. Outlook

#### 4.2.1. Using Less GWP-Intense Concrete

A main advantage of FRP reinforcement is its high resistance against corrosion. Because an alkaline environment is not required to protect the reinforcement from rusting, the concrete mix design can be optimised by reducing the clinker amount while simultaneously providing the same performance. Recent studies introduced mixes with up to 40% less cement clinker [66]. In the following, the influence of such less GWP-intense concretes is investigated by assuming the reduction in clinker corresponds to the reduction in overall GWP. This assumption is a simplification but is considered close to reality because the cement clinker accounts for >95% of the GWP in concrete [67].

Table 5 summarises the mass-related GWP for the individual structures for a conventional concrete mix design and an FRP-optimised mix design, where the strength and stiffness are considered to be the same. The given percentages are relative to the steel-reinforced structure. It can be seen that the mass-related GWP that stems from the local minimum in the optimum design curve is in all cases significantly lower than the steel-reinforced variant. It is also noticeable that the GWP of the reinforcement decreases although only the concrete mix design has changed. This indicates that the GWP minima shifts to lower reinforcement ratios and higher depths, so that the total mass increases.

#### 4.2.2. Outlook II: Decreasing Cost of Reinforcement

FRP reinforcement is still at a relatively early stage of development and applications are not widespread. It is often argued that with increasing demand for the reinforcement and increasing competition in the market, the price of FRP reinforcement will decrease. In this section, a decrease in costs of 30% is simulated and the effects on the total costs of the structure are compared, see Table 6. The given percentages are again relative to the steel variant for each structure.

It can be seen that, especially for the carbon-reinforced structures, a 30% decrease in the FRP reinforcement costs leads to a progressively larger decrease in the overall costs with increasing structure size. Interestingly, the costs of the concrete also decrease, meaning that the local minimum in the optimum design curve shifts towards a structure with a greater reinforcement area but a smaller depth of the structure. In all cases, however, the material costs of the FRP-reinforced structures are still greater than the steel-reinforced ones.

Immediate action that can be undertaken to lower the cost of the structure is to build hybrid-reinforced structures, e.g., to use FRP reinforcement only where the reinforcement is prone to corrosion. This is demonstrated in the following for the retaining wall example, where, due to pressurised water, the main reinforcement layer that was calculated in Section 3.2 is at particular risk of corrosion. Additional reinforcement to meet construction requirements is calculated to account for an additional 30% of the costs in the steel-reinforced variant (in total 386 EUR/m). If in the case of the FRP variants this additional reinforcement is provided using steel bars, the cost increase relative to the fully steel-reinforced structure can be cut by ~24% both for the GFRP and CFRP variants. The overall costs for the hybrid FRP-reinforced structures are then still higher compared to the fully steel-reinforced structure. However, a lower GWP at construction and a possible longer service life can be motivations for choosing an FRP variant over a fully steel-reinforced variant. In addition to enhancing the durability, a hybrid option with two layers of main reinforcement (one FRP layer and one steel layer) also improves the tensile response of a structure, as the stiffness is higher compared to a pure FRP-reinforced structure, while the steel reinforcement can be exploited in the post-yielding stage [68]. However, it should be noted that in hybrid steel–CFRP structures, direct contact between the CFRP and steel reinforcement must be avoided, as it triggers accelerated galvanic corrosion [69].

## 5. Conclusions and Outlook

In this paper, design curves for FRP-reinforced concrete structures under flexural conditions, that fulfil all stated requirements in the ULS (flexural capacity) and SLS (deflection control, crack-width limitations, concrete stress and reinforcement stress limits) were derived using parametric design studies. Subsequently, the mass-related GWP and material costs of different infrastructure buildings were studied. The influence of various parameters such as the size of the structure and concrete compressive strength on the environmental impact and economics was investigated. This allows for an evaluation of the potential for different types of FRP reinforcement in selected concrete infrastructure applications. The following general conclusions can be drawn:

- The optimum design curves for rectangular FRP-reinforced structures under flexural loading are generally driven by SLS requirements rather than the ULS flexural capacity. An optimised balance between concrete and FRP strength, which differs for the various structures under consideration, allows for higher overall utilisation rates.
- For the CFRP-reinforced structures, distinct local minima for cost or GWP exist, meaning that an optimised cross-sectional design has a decisive influence on both parameters. For the cases considered here, it was generally found to be favourable to have a larger concrete cross-sectional area and smaller CFRP reinforcement area. However, this would lead to a heavier structure.
- For the GFRP-reinforced structures, due to the lower environmental impact of the reinforcement, a smaller concrete cross-sectional area and larger reinforcement area was generally found to be more favourable in terms of the overall GWP of the structure.
- It is observed that FRP-reinforced structures are competitive with steel-reinforced options in terms of the GWP where, in particular, the GFRP-reinforced structures exhibit relatively low mass-related GWP. This trend is amplified if greater cement reductions in the concrete mixes can be realised due to the high corrosion resistance of FRP reinforcement.
- In terms of the mass-related costs, FRP-reinforced structures remain more expensive than steel-reinforced variants. This was still the case even when a potential price reduction of up to 30% due to mass production was simulated.

When assessing potential application areas for FRP reinforcement, the size of the structure plays a significant role, as follows:

- The smaller the structure, the smaller the difference in the material costs and GWP compared to steel structures. This observation can be traced back to the concrete cover having a more significant impact on the overall concrete volume in smaller structures. Hence, a reduction in the concrete cover due to the high corrosion resistance of the FRP reinforcement significantly reduces the GWP and costs of the overall structure. It is therefore more advantageous when the FRP textile reinforcement, which has a smaller reinforcement area and thus allows for a smaller wall thickness, is used in small structures. This type of reinforcement can also be more cost-efficient relative to FRP bars.
- Hybrid structures where only the reinforcement prone to corrosion is replaced by FRP reinforcement could suit medium-sized structures like the investigated retaining wall. A hybrid approach would lower the construction costs, while a structure with a lower GWP can still be achieved.
- Large-size FRP-reinforced structures such as bridges show significantly higher costs. This comparison was based on solid, reinforced cross sections and demonstrates that a simple replacement of steel reinforcement with FRP reinforcement, particularly CFRP, is not a suitable solution. Instead, a different design concept with more optimised, prestressed cross sections would take advantage of specific FRP reinforcement characteristics, such as the lower elongation stiffness, and thus better utilise the high-performance FRP reinforcement.

Using cost and energy demand due to production as the sole decision criterium does not capture other factors that are important when choosing the preferred design. The possible longer service life of an FRP-reinforced structure, combined with lower maintenance costs and higher material utilisation, would lead to economic and socio-political advantages. It is also noted that only rectangular cross sections were considered in this study. The potential for further optimisation exists, e.g., through cross-sectional or topology optimisation.

## Figures and Tables

**Table 1:** Input values of FRP reinforcement used in the parametric study.

	*f*_tk_^1^ (MPa)	*E*_r_ ^1^ (GPa)	*ρ*^1^ (t/m³)	*C*_T_ ^2^ (–)	*C*_C_ ^2^ (–)	*C*_E_ ^2^ (–)	*τ*_m_ ^3^ (MPa)	*w*_lim_ ^2^ (mm)	*γ*_m_ ^4^ (–)	GWP ^5^ (kgCO_2_ equ/kg)	Cost ^6^ (EUR/kg)
Carbon bar	2100	162	1.50	0.8	0.55	0.90	1.8	0.5	1.5	19.7	100
Carbon textile	3000	230	1.77		0.55	0.90			1.3	18.4	45
Glass bar	1300	59.5	2.13		0.35	0.70			1.5	3.1	8
Steel	550	200	7.86	−	−	−	1.8	0.3	1.15	2.3	1.0

^1^ Based on Reichenbach et al. [30]; ^2^ coefficients according to ACI 440-1R.15 [38]; ^3^ bond strength of wrapped FRP bars and epoxy-impregnated textiles can be of same magnitude as steel [40,41]; ^4^ according to preEN 1992-1-1 [42] and Rempel et al. [39]; ^5^ based on Stoiber et al. [4]; ^6^ data from German market [43,44]).

**Table 2:** Input values of concretes used in the parametric study.

	*f*_ck _ (MPa)	*f*_ctm_ (MPa)	*E*_cm_ (GPa)	*ε_cu2_* (‰)	*ρ* (t/m³)	*γ*_m_ (–)	GWP (kgCO_2_ equ/m³)	Cost (EUR/m³)
C20/25	20	2.2	30	3.5	2.4	1.5	178	100
C25/30	25	2.6	31				198.5	110
C30/37	30	2.9	33				219	120
C35/45	35	3.2	34				239.5	125
C40/50	40	3.5	35				260	135
C45/55	45	3.8	36				280.5	140
C50/60	50	4.1	37				300	145

**Table 3:** Optimum solutions in terms of the GWP and costs of the individual structures, broken down into steel or FRP reinforcement (denoted by ‘R’) and concrete (denoted by ‘C’) contributions. The percentage relates to the steel-reinforced variant of each structure.

		GWP Tot	GWP-R	GWP-C	Cost Tot	Cost-R	Cost-C
		(kg CO_2_ eq/m)	(kg CO_2_ eq/m)	(kg CO_2_ eq/m)	(EUR/m)	(EUR/m)	(EUR/m)
Rail Platform Barrier	Steel	29.2	7.7	21.5	15.3	3.4	11.9
	CFRP	25.7 (−11.9%)	5.6 (−27.9%)	20.2 (−6.1%)	20.9 (+36.6%)	7.6 (+125.2%)	13.3 (+11.6%)
	GFRP	26.4 (−9.7%)	5.1 (−34.3%)	21.3 (−0.9%)	20.9 (+36.6%)	7.3 (+116.8%)	13.6 (+14%)
Retaining wall	Steel	569.0	164.5	404.4	295.2	74.7	220.6
	CFRP	617.3 (+8.5%)	130 (−21%)	487.3 (+20.5%)	801.3 (+171.4%)	435 (+482.6%)	366.3 (+66.1%)
	GFRP	567.2 (−0.3%)	99.7 (−39.4%)	467.5 (+15.6%)	461.4 (+56.3%)	172.1 (+130.5%)	289.3 (+31.2%)
Integral Bridge	Steel	2459.0	846.1	1612.9	1251.6	367.8	883.8
	CFRP	2825.3 (+14.9%)	851 (+0.6%)	1974.3 (+22.4%)	5275.8 (+321.5%)	3870 (+952.1%)	1405.8 (+59.1%)
	GFRP	2470 (+0.4%)	594.3 (−29.8%)	1875.7 (+16.3%)	2232.6 (+78.4%)	1042.8 (+183.5%)	1189.8 (+34.6%)

**Table 4:** Optimum solutions in terms of the GWP and costs of the individual structures, broken down into steel or FRP reinforcement (denoted by ‘R’) and concrete (denoted by ‘C’) contributions. The percentage relates to the overall GWP and cost of each reinforced structure.

		GWP Tot	GWP-R	GWP-C	Cost Tot	Cost-R	Cost-C
		(kg CO_2_ eq/m)	(kg CO_2_ eq/m)	(kg CO_2_ eq/m)	(EUR/m)	(EUR/m)	(EUR/m)
Rail Platform Barrier	Steel	29.2	7.7 (26.4%)	21.5 (73.6%)	15.3	3.4 (22%)	11.9 (78%)
	CFRP	25.7	5.6 (21.6%)	20.2 (78.4%)	20.9	7.6 (36.3%)	13.3 (63.7%)
	GFRP	26.4	5.1 (19.3%)	21.3 (80.7%)	20.9	7.3 (34.9%)	13.6 (65.1%)
Retaining wall	Steel	569.0	164.5 (28.9%)	404.4 (71.1%)	295.2	74.7 (25.3%)	220.6 (74.7%)
	CFRP	617.3	130 (21.1%)	487.3 (78.9%)	801.3	435 (54.3%)	366.3 (45.7%)
	GFRP	567.2	99.7 (17.6%)	467.5 (82.4%)	461.4	172.1 (37.3%)	289.3 (62.7%)
Integral Bridge	Steel	2459.0	846.1 (34.4%)	1612.9 (65.6%)	1251.6	367.8 (29.4%)	883.8 (70.6%)
	CFRP	2825.3	851 (30.1%)	1974.3 (69.9%)	5275.8	3870 (73.4%)	1405.8 (26.6%)
	GFRP	2470.0	594.3 (24.1%)	1875.7 (75.9%)	2232.6	1042.8 (46.7%)	1189.8 (53.3%)

**Table 5:** Comparison of mass-related GWP for the individual structures with a conventional concrete mix or FRP-optimised concrete mix, broken down into steel or FRP reinforcement (denoted by ‘R’) and concrete (denoted by ‘C’) contributions. The percentage relates to the steel-reinforced variant of each structure.

		Conventional Concrete Mix Design			Concrete Mix Design with 40% Reduced GWP		
		GWP Tot	GWP-R	GWP-C	GWP Tot	GWP-R	GWP-C
		(kg CO_2_ eq/m)	(kg CO_2_ eq/m)	(kg CO_2_ eq/m)	(kg CO_2_ eq/m)	(kg CO_2_ eq/m)	(kg CO_2_ eq/m)
Rail Platform Barrier	Steel	29.2	7.7	21.5	−	−	−
	CFRP	25.7 (−11.9%)	5.6 (−27.9%)	20.2 (−6.1%)	17.2 (−41.2%)	3.7 (−52%)	13.5 (−37.4%)
	GFRP	26.4 (−9.7%)	5.1 (−34.3%)	21.3 (−0.9%)	17.5 (−40.3%)	3.2 (−58.6%)	14.3 (−33.7%)
Retaining wall	Steel	569.0	164.5	404.4	−	−	−
	CFRP	617.3 (+8.5%)	130 (−21%)	487.3 (+20.5%)	416.6 (−26.8%)	109.3 (−33.5%)	307.3 (−24%)
	GFRP	567.2 (−0.3%)	99.7 (−39.4%)	467.5 (+15.6%)	377.6 (−33.6%)	85.2 (−48.2%)	292.4 (−27.7%)
Integral Bridge	Steel	2459.0	846.1	1612.9	−	−	−
	CFRP	2825.3 (+14.9%)	851 (+0.6%)	1974.3 (+22.4%)	2035.6 (−17.2%)	851 (+0.6%)	1184.6 (−26.6%)
	GFRP	2470 (+0.4%)	594.3 (−29.8%)	1875.7 (+16.3%)	1695.2 (−31.1%)	431.8 (−49%)	1263.4 (−21.7%)

**Table 6:** Comparison of optimum mass-related costs for the individual structures with today’s costs of FRP reinforcement and a simulated cost reduction of 30%, respectively, broken down into steel or FRP reinforcement (denoted by ‘R’) and concrete (denoted by ‘C’) contributions. The percentage is relative to the steel-reinforced variant of each structure.

		FRP Costs as of Today			30% FRP Cost Reduction		
		Cost Tot	Cost-R	Cost-C	Cost Tot	Cost-R	Cost-C
		(EUR/m)	(EUR/m)	(EUR/m)	(EUR/m)	(EUR/m)	(EUR/m)
Rail Platform Barrier	Steel	15.3	3.4	11.9	−	−	−
	CFRP	20.9 (+36.6%)	7.6 (+125.2%)	13.3 (+11.6%)	18.6 (+21.6%)	5.8 (+73.4%)	12.7 (+7%)
	GFRP	20.9 (+36.6%)	7.3 (+116.8%)	13.6 (+14%)	18.7 (+22.3%)	5.1 (+51.8%)	13.6 (+14%)
Retaining wall	Steel	295.2	74.7	220.6	−	−	−
	CFRP	801.3 (+171.4%)	435 (+482.6%)	366.3 (+66.1%)	657.3 (+122.6%)	357 (+378.1%)	300.3 (+36.2%)
	GFRP	461.4 (+56.3%)	172.1 (+130.5%)	289.3 (+31.2%)	409.8 (+38.8%)	120.5 (+61.3%)	289.3 (+31.2%)
Integral Bridge	Steel	1251.6	367.8	883.8	−	−	−
	CFRP	5275.8 (+321.5%)	3870 (+952.1%)	1405.8 (+59.1%)	4069.8 (+225.2%)	2898 (+687.8%)	1171.8 (+32.6%)
	GFRP	2232.6 (+78.4%)	1042.8 (+183.5%)	1189.8 (+34.6%)	1919.8 (+53.4%)	730 (+98.4%)	1189.8 (+34.6%)

**Figure A1:**
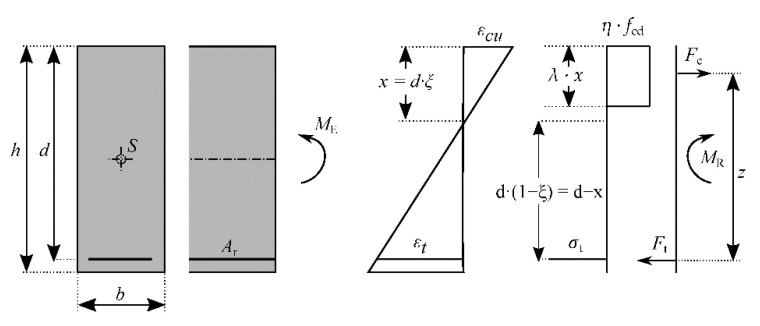
Calculation of flexural capacity *M*_R_ of an FRP-reinforced structure with an effective depth *d* reinforcement area *A*_r_ with a closed approach (assuming stress block of concrete compression zone with a height of λ · x); figure based on Rempel et al. [54]. (copyright 2022, John Wiley and Sons).

**Figure A2:**
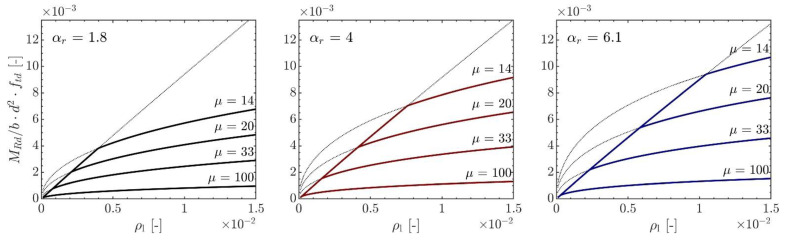
Normalised flexural capacity of reinforced concrete with different modulus ratio *α*_r_ and strength ratio *μ*.

**Figure A3:**
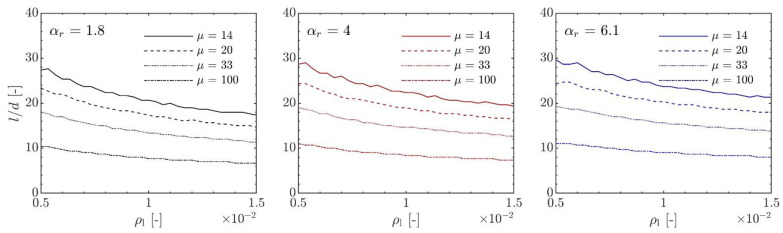
Possible *L/d* ratios where deflection control (*l*/250) of reinforced beams with different modulus ratio *α*_r_ and strength ratio *μ* is fulfilled. The deviations from a smooth line are the result of a limited number of increments in the effective depth and reinforcement ratio in order to reduce computational efforts.

**Figure A4:**
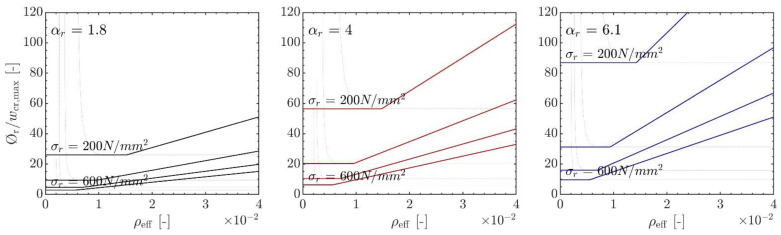
Normalised allowable crack width in dependency of effective reinforcement ratio *ρ*_eff_, ratio of modulus *α*_r_ and reinforcement stress σ_r_ (*τ*_m_ = 1.8 MPa, *k*_t_ = 0.4).

**Figure A5:**
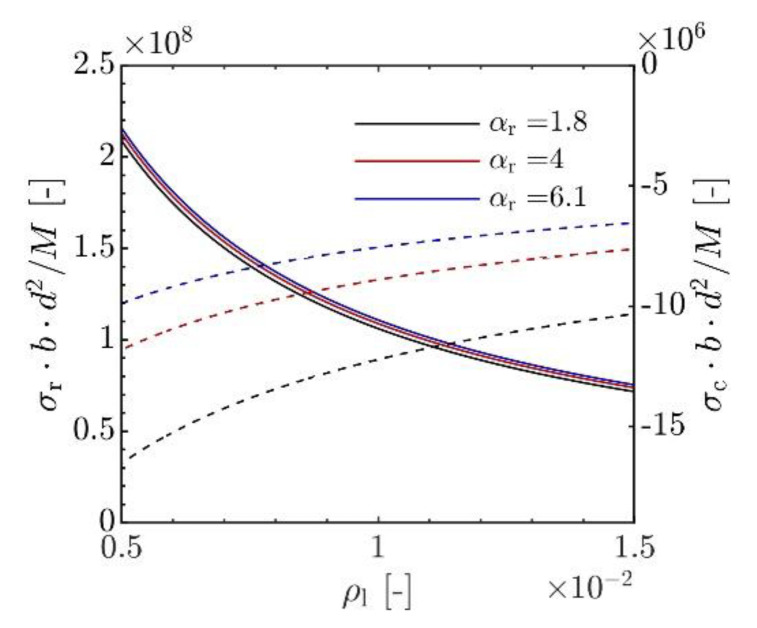
Normalised stresses in concrete and reinforcement under serviceability limit state in dependency of reinforcement ratio *ρ*_l_ and ratio of modulus *α*_r_.

**Figure A6:**
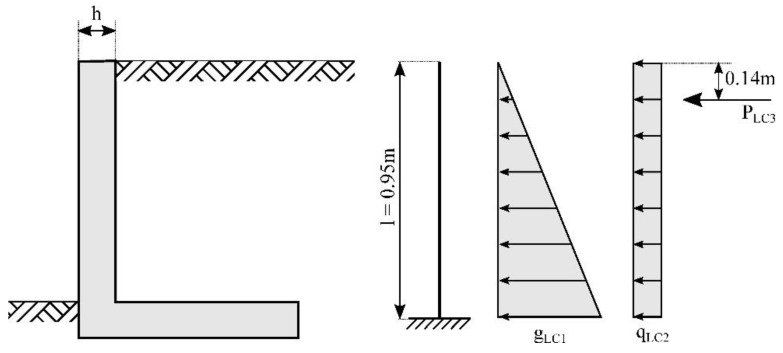
Loading conditions and static system of rail platform barrier.

**Figure A7:**
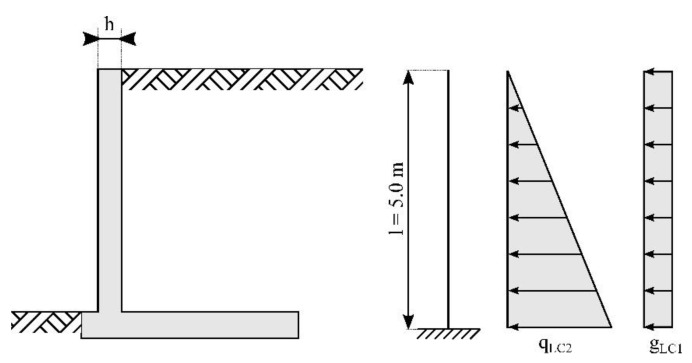
Loading conditions and static system of retaining wall.

**Figure A8:**
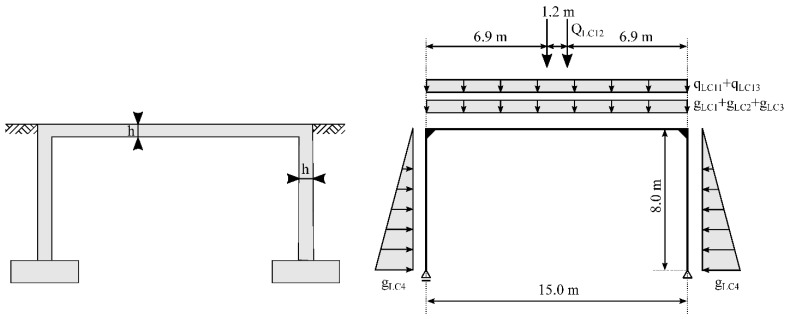
Loading conditions and static system of integral bridge.

## Data Availability

The data supporting the findings of this study will be made available on the repository of the University of Cambridge.

## Appendix A. Design Provisions

### Appendix A.1. Flexural Capacity

Assuming a stress block in the concrete compression zone (see Figure A1), the flexural capacity *M*_R_ of an FRP-reinforced structure with an effective depth *d* reinforcement area *A*_r_ can be calculated with a closed approach [54].

The balanced reinforcement ratio *ρ*_b_ where fibre rupture failure shifts towards concrete compression failure is calculated according to Equation (A1) with *η* and *λ* being 1.0 and 0.8, respectively, for concrete compressive strength *f*_ck_ < 50 MPa [45]:

(A1) $$\rho_{b}=\frac{f_{\mathrm{cd}}}{f_{\mathrm{td}}}\cdot \frac{\varepsilon_{\mathrm{cu}}}{\varepsilon_{\mathrm{tu}}+\varepsilon_{\mathrm{cu}}}\cdot \eta \cdot \lambda$$

with *ε*_tu_ and *ε*_cu_ being the ultimate strain at failure of the FRP and the concrete, respectively. In the case of *ρ*_l_ < *ρ*_b_, the flexural capacity is governed by rupture of the FRP reinforcement, which can be calculated using Equation (A2):

(A2) $$M_{\mathrm{Rd}}=\rho_{l}\cdot b\cdot d^{2}\cdot f_{td}\cdot \left(1-\frac{\rho_{l\cdot }f_{\mathrm{td}}}{\eta \cdot f_{\mathrm{cd}}}\right)$$

In the case of *ρ*_l_ > *ρ*_b_, flexural capacity is governed by concrete compression failure, which can be calculated using Equation (A3):

(A3) $$M_{\mathrm{Rd}}=\eta \cdot f_{\mathrm{cd}}\cdot b\cdot \lambda \cdot \xi \cdot d^{2}\cdot \left(1-\frac{\lambda \cdot \xi }{2}\right)$$

with *ξ* being the ratio of the concrete compression zone height *x* and the effective depth *d*:

(A4) $$\xi =\frac{x}{d}=\frac{\sqrt{\frac{E_{r}\cdot \varepsilon_{c}}{\eta \cdot f_{\mathrm{cd}}\cdot \lambda }}\cdot \sqrt{\frac{E_{r}\cdot \varepsilon_{c}}{\eta \cdot f_{\mathrm{cd}}\cdot \lambda }\cdot \rho_{l}+4}\cdot \sqrt{\rho_{l}}}{2}-\frac{\frac{E_{r}\cdot \varepsilon_{c}}{\eta \cdot f_{\mathrm{cd}}\cdot \lambda }\cdot \rho_{l}}{2}$$

Figure A2 depicts the normalised flexural capacity of reinforced structures over a broad range of parameters *α*_r_, *μ* and *ρ*_l_. The straight line marks the theoretical flexural capacity of the structure due to reinforcement rupture. After the balanced reinforcement ratio is reached, which is the point where the curves have a kink, the capacity is governed by nonlinear compressive failure.

### Appendix A.2. Deflection Control

To consider the partly cracked stage EN 1992-1-1 [45] suggests a distribution coefficient *ζ* between the uncracked *κ*_I_ and fully cracked *κ*_II_ stage, which is dependent upon the ratio of the reinforcement’s stress at first cracking *σ*_t,cr_ and the reinforcement’s stress under loading *σ*_t_:

(A5) $$\kappa =\zeta \kappa_{\mathrm{II}}+\left(1-\zeta \right)\kappa_{I}$$

with

(A6) $$\zeta =1-\beta \cdot \frac{\sigma_{t}}{\sigma_{t,\mathrm{cr}}}$$

For a given allowable deflection, possible *l/d* ratios that fulfil the serviceability requirement can be derived, as can be seen in Figure A3 for a simply supported beam with uniformly distributed loading (UDL). The allowable deflection is limited to *l/250* according to EN 1992-1-1. For a smaller reinforcement ratio *ρ*_l_ and smaller strength ratio *μ*, the *l/d* ratio potentially gets larger as the loading derived from the ultimate flexural capacity decreases. The modular ratio *α =E*_r_*/E*_c_, however, only plays a role for higher values of *ρ*_l_, where a bigger section of the beam is in a cracked state.

### Appendix A.3. Crack-Width Limitation

The crack width is calculated according to EN 1992-1-1, see Equation (A7):

(A7) $$w_{\mathrm{cr}}=s\cdot \left(\varepsilon_{r,m}-\varepsilon_{c,m}\right)$$

In the case of first cracking, the transfer length *I*_s_ is calculated at the point where strains in the reinforcement and concrete are equal. Assuming constant bond stress along the fibre strand, this results in Equation (A8):

(A8) $$w_{\mathrm{cr}}=\frac{\sigma_{r}\cdot {Ø}_{r}}{2\cdot \tau_{m}}\cdot \left(1-k_{t}\right)\cdot \frac{\sigma_{r}}{E_{r}}$$

with *k*_t_ being a factor to account for load duration (see also EN 1992-1-1 [45]), *σ*_t_ is the reinforcement stress under the relevant load combination and *Ø*_r_ is the reinforcement diameter. In the case of a stabilised crack pattern, the crack width is eventually calculated according to Equation (A9)

(A9) $$w_{\mathrm{cr}}=\frac{f_{\mathrm{ct}}\cdot {Ø}_{r}}{2\cdot \tau_{m}\cdot \rho_{\mathrm{eff}}}\cdot \left[\frac{\sigma_{r}}{E_{r}}-k_{t}\cdot \frac{f_{\mathrm{ct}}}{\rho_{\mathrm{eff}}\cdot E_{r}}\cdot \left(1+\alpha_{r}\cdot \rho_{\mathrm{eff}}\right)\right]$$

The normalised crack width is plotted in Figure A4 for different effective reinforcement ratio *ρ*_eff_, modular ratio *α*_r_ and stress in reinforcement *σ*_r_. It can be seen that the modular ratio has a direct influence on the crack width, which becomes even more evident in Figure A4.

In absence of more refined models for FRP-reinforced structures, the effective reinforcement ratio *ρ*_eff_ is calculated with an effective height of the tension chord *h*_c,eff_ according to EN 1992-1-1, see Equation (A10)

(A10) $$h_{c,eff}=min\left[2,5\cdot \left(h-d\right);\frac{h-x}{3};\frac{h}{2}\right]$$

### Appendix A.4. Stress Limitation

Considering a triangular stress distribution in the concrete, the stresses under serviceability limit state can be calculated according to Equations (A11) and (A12)

(A11) $$\xi =-\alpha_{r}\rho_{l}+\sqrt{{\left(\alpha_{r}\rho_{l}\right)}^{2}+2\alpha_{r}\rho_{l}}$$

(A12) $$\sigma_{c}=\frac{M_{E}}{bd^{2}\frac{\xi }{2}\left(1-\frac{\xi }{3}\right)}$$

(A13) $$\sigma_{r}=-\sigma_{c}\alpha_{r}\left(\frac{1}{\xi }-1\right)$$

The normalised stresses according to these equations are depicted in Figure A5 for varying reinforcement ratio *ρ*_l_ and modular ratio *α*.

## Appendix B

### Appendix B.1. Rail Platform Barrier

A rail platform barrier typically has an L-shape with the same thickness of the slab and the wall. For the calculations in this paper, the wall is simplified as a cantilever with a height of 0.95 m, see Figure A6.

The rail platform barrier is loaded by triangular earth pressure (LC1) from the backfilling. Additional earth pressure arises from gatherings of people (LC2) and a maintenance car that operates on the platform (LC3). The size of the loading and the respective combination and safety coefficients are listed in Table A1. Because it is a vertical element, no dead load due to self-weight is applied.

**Table A1:** Load cases that act on the rail platform barrier.

	LC1	LC2	LC3
*e*_k_ (kN/m)	9.50	2.50	
*E*_k_ (kN)			10.0
*ψ*_0_	1.00	0.70	1.00
*ψ*_2_	1.00	0.60	0.30
*γ*_D_	1.35	1.50	1.50

### Appendix B.2. Retaining Wall

Like the rail platform barrier, the retaining wall is L-shaped but larger in size, with a wall height of 5.0 m. The static system of the wall can again be described as a cantilever, see Figure A7.

The loadings arise from the triangular earth pressure of the backfill (LC1) and additional constant earth pressure due to a live load (LC2). Again, no dead load due to the self-weight of the wall is applied as it is a vertical element. The size of the loading and the respective combination and safety coefficients are listed in Table A2.

**Table A2:** Load cases that act on the retaining wall.

	LC1	LC2
ek (kN/m)	33.45	5.59
ψ0	1.00	1.00
ψ2	1.00	0.30
γD	1.35	1.50

### Appendix B.3. Integral Bridge

The bridge’s statical system is a frame with a height of 8.0 m and a length of 15.0 m. The cross-sectional height *h* of the horizontal and vertical frame elements is taken to be the same; see Figure A8.

The bridge is loaded by dead loads due to self-weight (LC1), the roadway structure (LC2 and LC3) and the earth pressure due to backfilling (LC4). Additional loads arise from the traffic load model LM1 as defined by EN 1992-2 [70]. It consists of a uniformly distributed load (LC11) and two point loads simulating a double-axle load (LC12). Finally, a uniformly distributed live load (LC13) is applied. The size of the loading and the respective combination and safety coefficients are listed in Table A3.

**Table A3:** Load cases that act on the integral bridge.

	LC1	LC2	LC3	LC4	LC11	LC12	LC13
*e*_k_ (kN/m)	Var.	2.80	3.04	40	3	−	2.22
*E*_k_ (kN)	−	−	−	−	−	33.3	−
*ψ*_0_	1.00	1.00	1.00	1.00	1.00	1.00	1.00
*ψ*_2_	1.00	1.00	1.00	1.00	0.4	0.75	0.4
*γ*_D_	1.35	1.35	1.35	1.00	1.35	1.35	1.35
